# DNA methylation loci associated with atopy and high serum IgE: a genome-wide application of recursive Random Forest feature selection

**DOI:** 10.1186/s13073-015-0213-8

**Published:** 2015-08-21

**Authors:** Todd M. Everson, Genevieve Lyons, Hongmei Zhang, Nelís Soto-Ramírez, Gabrielle A. Lockett, Veeresh K. Patil, Simon K. Merid, Cilla Sӧderhӓll, Erik Melén, John W. Holloway, S. Hasan Arshad, Wilfried Karmaus

**Affiliations:** Department of Epidemiology and Biostatistics, Arnold School of Public Health, University of South Carolina, 915 Greene Street, Columbia, SC 29208 USA; Department of Biostatistics, University of Texas M. D. Anderson Cancer Center, Pickens Tower, 1400 Pressler, Houston, TX 77230 USA; Division of Epidemiology, Biostatistics and Environmental Health, School of Public Health, University of Memphis, 236A Robison Hall, Memphis, TN 38152 USA; Human Development and Health, Faculty of Medicine, University of Southampton, Southampton General Hospital, Southampton, SO16 6YD UK; The David Hide Asthma and Allergy Research Centre, St Mary’s, Hospital, Parkhurst Road, Newport, Isle of Wight PO30 5TG UK; Institute of Environmental Medicine, Karolinska Institutet, Stockholm, Sweden; Department of Biosciences and Nutrition, and Center for Innovative Medicine (CIMED), Karolinska Institutet, 141 83 Stockholm, Sweden; Sachs’ Children’s Hospital, Stockholm, Sweden; Clinical and Experimental Sciences and NIHR Respiratory Biomedical Research Unit, Faculty of Medicine, University of Southampton, Southampton General Hospital, Southampton, SO16 6YD UK

## Abstract

**Background:**

The prevalence of allergic diseases are increasing worldwide, emphasizing the need to elucidate their pathogeneses. The aims of this study were to use a two-stage design to identify DNA methylation levels at cytosine–phosphate–guanine (CpG) sites across the genome associated with atopy and high serum immunoglobulin E (IgE), then to replicate our findings in an independent cohort.

**Methods:**

Atopy was assessed via skin prick tests and high serum IgE. Methylation levels were measured from whole blood using the Illumina Infinium HumanMethylation450 BeadChip from 18-year-old women (n = 245) and men (n = 122) in the Isle of Wight birth cohort. After data cleaning and processing, and removing probes with possible single nucleotide polymorphisms, DNA methylation levels from 254,460 CpG sites from the 245 women were subjected to recursive Random Forest feature selection for stage 1. The sites selected from stage 1 were tested in stage 2 for associations with atopy and high IgE levels (>200 kU/L) via logistic regression adjusted for predicted cell-type proportions and sex. Sites significantly associated with atopy in stage 2 underwent replication tests in the independent Swedish birth cohort BAMSE (n = 464).

**Results:**

In stage 1, 62 sites were selected, of which 22 were associated with atopy in stage 2 (*P*-value range 6.5E−9 to 1.4E−5) and 12 associated with high IgE levels (*P*-value range 1.1E−5 to 7.1E−4) at the Bonferroni adjusted alpha (0.05/62 = 0.0008). Of the 19 available sites, 13 were replicated.

**Conclusions:**

We identified 13 novel epigenetic loci associated with atopy and high IgE that could serve as candidate loci for future studies; four were within genes with known roles in the immune response (cg04983687 in the body of *ZFPM1*, cg18219873 in the 5′UTR of *PRG2*, cg27469152 in the 3′UTR of *EPX*, and cg09332506 in the body of *COPA*).

**Electronic supplementary material:**

The online version of this article (doi:10.1186/s13073-015-0213-8) contains supplementary material, which is available to authorized users.

## Background

The prevalence of allergic disease is increasing worldwide; approximately 40 % of the population of industrially developed countries are considered to be affected [1]. Many of these allergic diseases appear to have a hereditary component but are also influenced by environmental stimuli [2], and the origin of the immune response, including allergen sensitization, is thought to start during the fetal period [3]. It is well recognized that environmental stimuli during critical prenatal and postnatal periods can permanently alter metabolism and influence the risk of allergic diseases [4], yet the specific molecular mechanisms through which this occurs are poorly understood [1, 5].

Epigenetics, changes in gene activity not caused by alterations to the sequence of DNA, may clarify some of these mechanisms because much of cell lineage and tissue-specific gene expression is tightly regulated by epigenetic programming [1]. One of the most commonly studied epigenetic mechanisms is DNA methylation (DNA-M), the covalent addition of a methyl group to a cytosine followed by a guanine (cytosine–phosphate–guanine; CpG). Changes in DNA-M affect gene transcription and have been associated with disease [6]. Some of DNA-M’s roles in the development of the immune system, immune cell-fate, and allergic diseases have been unlocked, but substantial gaps in knowledge still exist [1].

Atopy is defined as a positive reaction to a skin prick test (SPT) or immunoglobulin E (IgE) production in response to allergens [7]. IgE plays an important role in many, but not all, allergic diseases, for example, asthma, rhinitis, and eczema [7, 8]. High levels of IgE in the blood are associated with both the risk and severity of asthma, and cord blood IgE levels have been studied as possible predictors of asthma and other atopic allergic diseases [4]. Atopy is therefore connected to allergic disease, although many of the details of this relationship are still unknown. Epigenetic epidemiology can help to clarify the role that DNA-M plays in atopy by confirming candidate loci and revealing novel loci associated with atopy [5].

Advances in genetic biotechnology have made it feasible to measure DNA-M throughout an individual’s epigenome and, consequently, epigenetic assessments are becoming feasible in larger epidemiologic studies [9]. A growing challenge with epigenetic epidemiology is that a vast amount of data is generated and new statistical techniques are necessary to make sense of it. This is because of small-*n*-large-*p* (few observations relative to the number of predictors) and because traditional methods are not optimized for identifying complex biological processes. Because of the large-scale data generated for each completed methylation assay, techniques to select a subset of informative variables are needed [10–12], particularly in cases of sparse data in which the vast majority of predictors are uninformative [13].

Random Forest (RF), developed by Leo Breiman, is a machine learning algorithm used for classification that can handle the data issues discussed above [14]. A forest composed of classification trees is grown using randomly selected bootstrap samples of the data to form training and testing sets of study participants. At each node within each tree, the training set is partitioned into different classes with the split determined by a subset of randomly chosen predictors. These two levels of randomness, random selection of training/testing sets and random testing of predictors, allow the RF to produce robust classification predictions. Once the forest is grown using the training sets, the observations in the testing sets are classified via the forest and misclassification rates can be used to evaluate the accuracy of the forest [14].

Utilization of RF to analyze array data has increased in recent years [15–18]; it is an ideal method for classification with methylation data for several reasons. Unlike most traditional methods, RF can be used for feature selection when the number of variables exceeds the number of observations, even when most variables are uninformative; RF can use both numeric and categorical variables; the algorithm can be optimized fairly easily by varying the RF parameters; and adding trees does not cause the model to become over-fit [14, 15, 19]. In addition, biological processes are probably not linear in nature; rather, they involve interactions between many different molecules. Thus it is likely that methylation changes at a combination of CpG sites could influence disease states. RF allows for the identification of multiple interacting predictors and identifies which of these are most important without imposing a structure or model on the way that it takes place.

Despite its increased presence in the analysis of genomic data, few applications of the algorithm have utilized the variable importance measures (VIM) and its potential for feature selection [18]. While RF lends itself to a variety of applications, we focused on using it for feature selection.

In this study, we implemented a two-stage discovery study within the Isle of Wight (IOW) birth cohort to first select a set of atopy-candidate CpGs from epigenome-wide data using a subsample and then to test which of these sites were significantly associated with atopy as defined by positive SPT or high total serum IgE levels in the joint sample. Then, to validate our findings, we ran replication tests in the independent Swedish cohort BAMSE.

## Methods

### The Isle of Wight birth cohort

The IOW birth cohort was established to study the natural history of allergic disease among children born between 1 January 1989 and 28 February 1990 on the Isle of Wight, UK. The study was approved by the local research ethics committee (now named the National Research Ethics Service, NRES Committee South Central – Southampton B; 06/Q1701/34) and written informed consent was provided by the infants’ parents. After exclusion of adoptions, perinatal deaths, and refusals, 1,456 children (95 %) were enrolled. Participants were followed-up at ages 1 (n = 1,167), 2 (n = 1,174), 4 (n = 1,218), 10 (n = 1,373), and 18 years (n = 1,313); detailed questionnaires were administered at each follow-up. Details of the birth cohort have been described elsewhere [20, 21]. At age 18 years, 245 women and 122 men were randomly selected from the cohort for genome-wide DNA methylation screening as part of another study assessing trans-generational inheritance of atopy.

### Data collection and DNA methylation

At the 18-year follow-up, most of those who were seen in-person received SPTs using a standard method [22] and with a battery of common allergens. Inhalant allergens tested were house dust mite, cat, dog, *Alternaria alternata*, *Cladosporium herbarum*, grass pollen mix, and tree pollen mix. Food allergens tested were cows’ milk, soy, hens’ egg, peanut, and cod. Of the 245 women included in the epigenetic analyses, SPTs were conducted on 242 of them; 120 of the men received SPTs. Blood samples for DNA extraction and serum for measurements of IgE levels were also collected at 18 years of age. Total serum IgE was measured in a subset of male and female serum samples collected at age 18 (n = 144) using Immunocap (Phadia, Uppsala, Sweden), designed to measure IgE between 2.0 to 1,000 kU/L. DNA was extracted from whole blood using a standard salting-out procedure [23]. DNA concentration was determined by the Qubit quantitation kit (Life Technologies Ltd, Paisley, Renfrewshire, UK). One microgram of DNA was bisulfite-treated for cytosine to thymine conversion using the EZ 96-DNA methylation kit (Zymo Research, Irvine, CA, USA), following the manufacturer’s standard protocol. Genome-wide DNA methylation was assessed using the Illumina Infinium HumanMethylation450K BeadChip (Illumina, Inc., San Diego, CA, USA), which interrogates >484,000 CpG sites associated with approximately 24,000 genes. The BeadChips were scanned using a BeadStation, and the methylation levels (β value, described below) were calculated for each queried CpG locus using the methylation module of GenomeStudio software (Illumina, Inc.). Arrays were processed using a standard protocol as described elsewhere [24], with multiple identical control samples assigned to each batch to assess assay variability and samples randomly distributed on microarrays to control against batch effects.

### Data cleaning

The program for data cleaning was written in R (R Development Core Team, 2012). Quality control (QC) measures were employed to improve the reliability of data prior to analysis. In our study, the detection *P*-value reported by GenomeStudio was used as a QC measure of probe performance. Probes with detection *P*-values > 0.01 in >10 % of the samples were removed [25]. The methylation data were then preprocessed and technical variations removed via peak-correction using the Bioconductor IMA (Illumina Methylation Analyzer) package. Excluding control probes and probes with poor detection *P*-values yielded 383,998 remaining probes; 9,650 CpGs on the sex chromosomes were also removed. The arrays were processed in two batches; batch number was recorded as a categorical variable, which was used in ComBat to adjust for inter-array variation [26, 27]. Because the female and male samples were assessed in different batches, some sites that survived QC in the female sample did not survive QC in the male sample. A very conservative approach was utilized for addressing intra-probe single nucleotide polymorphisms (SNPs); to ensure that our findings were not biased by SNPs affecting methylation levels, we excluded all probes with potential SNPs in the binding region or at base-pair extension (119,888 probes) according to the dbSNP database (version 137), resulting in a final set of 254,460 CpGs for analysis. Removing all probes with possible SNPs was necessary with our variable selection method because the selection of any variable is conditional upon the effects of other selected variables, thus inclusion of SNP-biased probes can affect the inclusion of other unbiased probes.

### Variable definitions

Participants were defined as being atopic, the primary outcome variable for this study, if they had a positive SPT to at least one of the tested allergens [7]. Positive SPTs were determined by a mean wheal diameter of 3 mm greater than the negative control; SPT results were deemed inconclusive if the positive control resulted in a diameter less than 3 mm. To internally validate our findings from the SPT analyses, we also tested the same statistical models but with dichotomous serum IgE levels (IgE ≥ 200 kU/L versus IgE < 200 kU/L), which has been shown to be predictive of allergy [28], as an alternate outcome variable.

Methylation levels for each queried CpG were calculated as β values. These represent the proportions of methylated (M) over methylated (M) and unmethylated (U) sites (β = M/[c + M + U], with constant c introduced to prevent the possibility of a zero in the denominator), and can be interpreted as percent methylation; β values close to 0 or 1 tend to suffer from severe heteroscedasticity. The β values were utilized for RF, described below, which is a non-parametric method and does not assume a normal distribution. However, for parametric statistical analyses, such as logistic regressions used for validation and replication, we utilized M-values, which address the issue of heteroscedasticity and thus perform better. M-values were calculated from the β values via log_2_[β/(1 − β)] [29]. Prior to running parametric models, boxplots and histograms were used to verify approximate normality and identify potential outliers.

Pearson’s chi-squared tests were used to determine if prevalence of atopy and high IgE differed between the female and male samples, within the epigenetic sample, and between the epigenetic sample and the entire cohort. *P*-values were compared against an α-level of 0.05. We implemented a two-stage genome-wide approach [30]: stage 1 analyses selected a set of atopy-candidate loci from genome-wide DNA-M within a subsample (n = 245), and stage 2 analyses tested those loci for associations with atopy and an alternate marker of atopy, high IgE, in the joint sample (n = 367). The specific methods within each stage are detailed below. The normalized DNA-M microarray data, as well as covariates and outcomes used in both stage 1 and stage 2 analyses, described below, are available via the University of Southampton ePrints Soton (DOI: 10.5258/SOTON/379389).

### The BAMSE cohort

Sites that were significantly associated with atopy in stage 2 analyses were selected for replication in the Children, Allergy, Milieu, Stockholm, Epidemiology (BAMSE), a prospective population-based cohort study of children recruited at birth and followed during childhood. Details of the study design, inclusion criteria, enrolment, and data collection are described elsewhere [31]. In brief, 4,089 children born between 1994 and 1996 in four municipalities of Stockholm County were enrolled. Longitudinal sensitization and questionnaire data were collected through to age 8. The baseline and follow-up studies were approved by the Regional Ethical Review Board, Karolinska Institutet, Stockholm, Sweden, and the parents of all participating children provided informed consent. Blood samples collected at 8 years were screened with Phadiatop [a mixture of common inhalant allergens: birch, timothy, mugwort, cat, dog, horse, mold (*Cladosporium herbarum*), and house dust mite (*Dermatophagoides pteronyssinus*)] and fx5 (a mixture of common food allergens: cow’s milk, egg white, soy bean, peanut, cod fish, and wheat) (ImmunoCAP, Phadia AB, Uppsala, Sweden). Atopy was defined as a positive Phadiatop or a positive fx5 test with specific IgE antibody levels ≥0.35 kUA/L. Furthermore, epigenome-wide DNA methylation was measured in 472 children using DNA extracted from blood samples collected at the 8 year follow-up [32]. For this, 500 ng DNA per sample underwent bisulfite conversion using the EZ-96 DNA Methylation kit (Shallow; Zymo Research Corporation, Irvine, CA, USA). Samples were processed with the Illumina Infinium HumanMethylation450 BeadChip (Illumina, Inc.). Data pre-processing (signal correction and data normalization) and QC were performed using standard criteria described elsewhere [33]. This study included those with valid DNA-M samples and that were non-missing for atopy-status or adjustment covariates (N = 464).

### Statistical analysis (IOW) – stage 1

The randomForest package in R was used to implement the RF algorithm [34]. The output from the RF includes the out-of-bag error rate (OOB-ER), class-specific misclassification rates, and VIMs. The OOB-ER is the overall misclassification rate of the complete forest. Class-specific misclassification rates, which are also calculated from the out-of-bag samples, are the rates at which the classes of the outcome variable are misclassified, in our case atopic classification and non-atopic classification. VIMs are measures of the amount of information a variable contributed to the classification throughout the forest. Hapfelmeier and Ulm, whose proposed feature selection method used OOB-ER or another cross-validated error measure, acknowledge that the VIM depends on the data and the underlying research question [35]. We used the mean decrease Gini (MDG) as VIM because it was shown to be more robust to small deviations to the data when compared to the mean decrease accuracy (MDA) [36].

Prior to implementing the recursive RF [15] described below, we explored how prediction accuracy of the forest was influenced by altering the parameters *sampsize*, *mtry*, and *ntree*, so these could be optimally set for the recursive RF implementation described below. The *sampsize* parameter controls whether to use balanced or imbalanced sampling to generate the training datasets; *mtry* specifies the number of variables to be randomly selected and tested at each node of each tree; and *ntree* determines the number of trees to be grown in a forest. Using the default values for *mtry* (√p, where p is the number of variables available) and *ntree* (500), we compared the OOB-ER and class-specific misclassification rates for an imbalanced RF grown without *sampsize* and a balanced RF grown with *sampsize* = (50,50). Specifying *sampsize* = (50,50) meant that 50 observations from those with atopy and 50 observations from those without atopy were randomly selected when creating the training set for each tree. Once we determined whether or not to utilize the *sampsize* parameter, we tracked the prediction accuracy of the RF at different combinations of *mtry* (√p, 2*√p, 0.05p, 0.1p, and 0.5p) and *ntree* (200, 300, 400, 500, 1,000, and 2,000). Once the optimal parameter values were selected, the recursive RF was implemented.

**Fig. 1:**
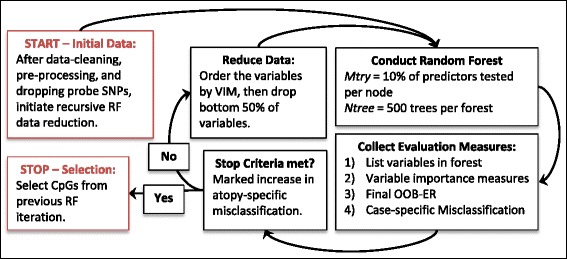
Recursive RF feature selection process. The feature selection process started with a large dataset: all CpGs that survived data cleaning and preprocessing, and were not potentially affected by probe SNPs. The cycle in *black* (conducting the Random Forest, collecting evaluation measures, assessing stop criteria, and reducing the data) repeated until the atopy-specific misclassification rate showed a marked increase, indicating that some excluded sites were important in classifying atopic participants. Thus, once an increase in atopy-specific misclassification was observed, the cycle stopped and sites from the previous iteration were selected for follow-up testing. *OOB-ER* out-of-bag error rate, *RF* Random Forest, *VIM* variable importance measure

The general methodology of the recursive RF for feature selection has been proposed and utilized elsewhere [13, 15, 17, 18, 35, 37], though not with high-throughput epigenetic data. Using this approach we aimed to reduce the data from all CpG sites retained after pre-processing and cleaning to a more manageable size by eliminating variables that contributed little predictive information for atopy. The recursive RF loop was initiated by running a RF with all CpGs included as potential predictors. Then the variables were sorted by their VIM, the bottom half of the CpGs with the lowest VIMs were removed, and the RF was run again, using this subset of CpG sites (Fig. 1). This process was repeated while tracking the RF OOB-ER and class-specific misclassification rates at each iteration. The process was stopped when the atopy-specific misclassification rate increased, because we were most concerned with correct classification of those with atopy. The variables from the iteration prior to the increase in misclassification were selected for further analyses. Each CpG site that was selected by the recursive RF was annotated with information about what gene the CpG site was within, when applicable.

The sites selected from the recursive RF were then tested for univariate associations with atopy via logistic regression. Given that methylation levels vary significantly by cell type, peripheral blood samples are composed of multiple different cell types, and allergic diseases often influence the proportions of circulating immune cell types, we considered the potential confounding effect of cell-type differential for each participant. However, logistical limitations prevented the acquisition of cell differential at the original time of blood sample collection. Thus, we utilized the methylation data to predict cell differential [38, 39], then calculated the percent differences between the crude and cell-type adjusted β coefficients from the logistic regression models, to observe the impact of cell type on the association between methylation levels and atopy.

### Statistical analysis (IOW) – stage 2

Boxplots of β values stratified by atopy status were used to ensure that the distributions of methylations levels in the female and male samples were similar and could be combined. Two observations (in cg12819873 and in cg13168187) were identified as strong outliers, and re-coded as missing. The joint sample was then used for all stage 2 analyses in which each CpG was tested for its individual association with atopy and high IgE, adjusted for important covariates. These tests were conducted with logistic regression in which high IgE and atopy were the dependent variables and M-values for CpGs were the primary independent variables, while cell type proportions and sex were included as covariates. CpG sites that were significantly associated with both atopy and high IgE at the Bonferroni corrected α were subjected to set analyses, used to show the combined effect of DNA-M on atopy [40].

### Statistical analyses (BAMSE) – replication

For each site that was significantly associated with atopy in stage 2, we conducted multiple logistic regression models in the BAMSE cohort. Atopy status was the dependent variable and M-values for CpGs were the independent variables, while cell type proportions, sex, asthma treatment within the last 12 months, and batch (bisulfite treatment date) were included as covariates (identified as confounders in the regression model). Successful replication was defined as having the same direction of association and a *P*-value < 0.05. These sites were submitted for functional annotation analyses in DAVID [41, 42].

## Results

All IOW participants were age 18 years at the time of epigenetic screening for DNA-M and administration of SPTs. Of all participants that underwent epigenetic screening, three females and two males did not receive SPTs and thus were not assessed for atopy status. Although there were some differences in the prevalence of atopy and high IgE between the epigenetic subsample and the full cohort, none of these differences were statistically significant. The prevalence of atopy within the epigenetic subsample was not significantly different (*P*-value = 0.0972) between the female sample, used in stage 1, and the male sample which was pooled with the female sample in stage 2. However, prevalence of high serum IgE (≥200 kU/L) was significantly (*P*-value = 0.0469) lower in the female sample (23.8 %) than in the male sample (41.9 %). These differences in high IgE did not affect the analytic methods because serum IgE was only used as a secondary and alternate biomarker of atopy to provide further confidence in our top results (Table 1).

**Table 1:** Prevalence of atopy via positive skin prick tests and high serum IgE among females and males

		Epigenetic subsample			Full cohort		Subsample vs full cohort
Outcome variables		Females	Males	*χ* ^2^	Females	Males	*χ* ^2^
		(n = 245)	(n = 122)	*P*-value	(n = 786)	(n = 750)	*P*-value
Atopy	At least one positive	76 (31.4 %)	49 (40.8 %)	0.097	159 (35.6 %)	194 (47.7 %)	Females: 0.30
	All negative	166 (68.6 %)	71 (59.2 %)		287 (64.4 %)	213 (52.3 %)	Males: 0.22
IgE	IgE ≥ 200 kU/L	24 (23.8 %)	18 (41.9 %)	0.047	66 (25.9 %)	81 (32.5 %)	Females: 0.78
	IgE < 200 kU/L	77 (76.2 %)	25 (58.1 %)		189 (74.1 %)	168 (67.5 %)	Males: 0.31

For stage 1 we conducted a recursive RF algorithm with feature selection very similar to balanced iterative RF, described in detail in the methods section [18]. Prior to implementing the full algorithm we optimized the RF parameters by testing multiple combinations *mtry* and *ntree*. We selected an *mtry* of 0.1p, which was observed to be effective in a similarly large scale RF analysis with sparsity [17], and an *ntree* of 500 that allowed the error rates to stabilize, but limited computational time.

The initial RF in the recursive implementation was fitted with all CpG sites (p = 254,460) that survived data cleaning, pre-processing, and removal of probes potentially containing SNPs. At each step in the reduction, the dataset was reduced by half; by the 15th iteration the data was reduced to a total of 15 CpG sites. The OOB-ER achieved its lowest point (overall misclassification of 8.67 %) at the 11th iteration, which included 248 CpGs (Table 2). However, we reduced the data further to the 13th iteration, which resulted in the lowest misclassification of atopics (14.47 %) and included 62 CpGs. From the first iteration to the 13th, the OOB-ER improved from 38.42 to 9.50 %, while the misclassification for atopics and non-atopics improved from 78.95 to 14.47 % and 19.87 to 7.22 % respectively. After the 13th iteration, each of the misclassification rates increased, thus the CpGs (p = 62) from this iteration were selected for stage 2 analyses.

**Table 2:** Misclassification rates throughout the recursive RF process

Iteration	Number of variables	OOB-ER overall misclassification (%)	Non-atopic misclassification (%)	Atopic misclassification (%)
1	254,460	38.43	19.87	78.95
2	127,230	35.12	17.46	73.68
3	63,615	33.05	19.27	63.15
4	31,807	27.68	10.24	65.78
5	15,903	24.38	9.03	57.89
6	7,951	16.94	4.21	44.73
7	3,975	14.87	5.42	35.52
8	1,987	11.15	4.21	26.31
9	993	11.57	4.81	26.31
10	496	9.09	5.42	17.10
11	248	8.67	5.42	15.78
12	124	9.09	5.42	17.10
13	62	9.50	7.22	14.47
14	31	11.98	9.63	17.10
15	15	15.70	13.85	19.73

*OOB-ER* out-of-bag error rate

All 62 selected CpG sites were annotated with relevant genetic information (Table 3). We used logistic regression to describe the individual associations of all the selected CpG sites. Only cg09570585 and cg10016610 had *P*-values > 0.05 (*P*-values = 0.06353 and 0.09771, respectively). Prior to implementing stage 2 analyses, we tested whether any of the selected sites may have been selected due to confounding by cell type. Many of the associations were altered by adjusting for proportions of CD8^+^ T cells, CD4^+^ T cells, natural killer cells, B cells, monocytes, and granulocytes (Table 4). Thus all further associations were adjusted for cell type.

**Table 3:** Genetic annotations for 62 sites selected by recursive Random Forest

CpG Site	Chr	Coordinate	Associated genes	Gene region	CpG island
cg00854799	1	2336398	*PEX10*; *RER1*	3′UTR	North Shelf
cg09249800	1	6341287	*ACOT7*	Body	Island
cg06824199	1	47157809	*KIAA0494*	Body	–
cg17594242	1	115654782	*–*	–	–
cg09332506	1	160309220	*COPA*	Body	North Shelf
cg01847596	2	95660093	*–*	–	North Shelf
cg07880854	2	112895559	*FBLN7*	TSS1500	North Shore
cg13168187	2	159523681	*PKP4*	Body	–
cg01203365	2	217291500	*SMARCAL1*	Body	–
cg27468224	4	55031503	*–*	–	–
cg11372831	4	57303157	*PAICS*; *PPAT*	Body; TSS1500	South Shore
cg03553407	4	148863880	*ARHGAP10*	Body	–
cg00528600	5	61699751	*DIMT1L*	TSS200	Island
cg04085542	5	93414338	*FAM172A*	Body; 5′UTR	–
cg05560165	5	133450315	*TCF7*	TSS1500	Island
cg14322298	6	10585683	*GCNT2*	Body; TSS1500	–
cg03131171	6	37616686	*MDGA1*	Body	North Shore
cg00155310	6	50814011	*TFAP2B*	3′UTR	South Shore
cg02201050	7	22759083	*–*	–	–
cg02366798	7	27237154	*HOXA13*	3′UTR	North Shore
cg09570585	7	138916241	*UBN2*	1stExon	Island
cg05652668	7	139044807	*LUC7L2*	1stExon; 5′′UTR	Island
cg07970948	7	149543165	*ZNF862*	Body	–
cg24836822	7	150648840	*KCNH2*	Body	Island
cg05104993	8	11973223	*FAM66D*	TSS200	North Shore
cg06816054	8	27695695	*PBK*	TSS1500	South Shore
cg04775941	8	141474793	*–*	–	Island
cg13713293	9	841636	*DMRT1*	TSS200	Island
cg25854298	10	73936754	*ASCC1*	Body	–
cg03468115	10	76852752	*–*	–	–
cg23527183	10	95253833	*–*	–	North Shelf
cg08397758	10	100174853	*PYROXD2*	1stExon	–
cg06851336	10	104678166	*CNNM2*	1stExon; 5′UTR	Island
cg24077454	10	119134782	*PDZD8*	1stExon; 5′UTR	Island
cg14574726	11	809735	*RPLP2*	TSS200	Island
cg12819873	11	57157632	*PRG2*	5′UTR	–
cg13233042	11	63432489	*ATL3*	Body	–
cg04162999	11	64120313	*CCDC88B*	Body	–
cg10016610	11	124735994	*ROBO3*	Body	Island
cg07908654	13	41631052	*–*	–	North Shelf
cg09635874	13	98952518	*FARP1*	Body	–
cg11182893	13	114842103	*RASA3*	Body	South Shore
cg14478663	14	51643693	*–*	–	–
cg15281774	15	73661908	*HCN4*	TSS1500	Island
cg01777765	16	1823191	*MRPS34*; *NME3*; *EME2*	TSS200; TSS1500	Island
cg05048002	16	30077837	*ALDOA*	5'UTR	Island
cg04342090	16	30670571	*–*	–	Island
cg02775369	16	56316221	*GNAO1*	Body	–
cg01190915	16	56642761	*MT2A*	Body	South Shore
cg04983687	16	88558223	*ZFPM1*	Body	Island
cg27202913	16	89258862	*CDH15*	Body	Island
cg01097406	16	89675127	*–*	–	–
cg04798929	17	8287246	*RPL26*	TSS1500	Island
cg17549513	17	9694789	*DHRS7C*	TSS200	–
cg07765167	17	36451845	*MRPL45*	TSS1500	North Shore
cg27469152	17	56282313	*EPX*	3′UTR	–
cg17041511	17	61509620	*–*	–	North Shelf
cg12819826	19	10216676	*PPAN*; *PPAN–P2RY11*	TSS1500; TSS1500	North Shore
cg12578575	19	54135140	*DPRX*	TSS200	–
cg11569718	19	58905979	*RPS5*	Body	North Shore
cg13197551	20	60709957	*LSM14B*	3′UTR	–
cg17971837	22	37215996	*PVALB*	TSS1500	South Shelf

*Abbreviations: Chr* Chromosome number, *CpG* cytosine–phosphate–guanine*IgE* Immunoglobulin E, *IOW* Isle of Wight cohort, *TSS* Transcription Start Site, *UTR* untranslated region

Coordinate: Location of the CpG site within each chromosome, via genome build 37

**Table 4:** Stage 1 – Assessment of the influence of cell type on CpG selection in stage 1 analyses (n = 245)

CpG Site	Crude β_1_	Crude *P*-value	Adjusted β_1_	Adjusted *P*-value	%Diff_β_
cg00155310	2.24	0.00066	2.24	0.0011	−0.26
cg00528600	1.66	0.0038	1.68	0.0043	1.11
cg00854799	1.23	0.0062	1.25	0.0067	1.29
cg01097406	0.25	0.0050	0.27	0.0042	5.08
cg01190915	2.43	0.00020	2.61	0.00011	7.49
cg01203365	−1.25	0.0051	−1.19	0.0087	−4.59
cg01777765	−1.51	0.0030	−1.69	0.0015	11.76
cg01847596	−2.62	0.0015	−2.65	0.0019	1.49
cg02201050	2.08	0.0012	2.10	0.0017	0.87
cg02366798	0.84	0.0097	0.85	0.014	0.46
cg02775369	−1.55	0.00072	−1.53	0.0011	−1.37
cg03131171	−1.21	0.0064	−1.28	0.0049	5.47
cg03468115	1.68	0.0017	1.58	0.0052	−5.88
cg03553407	1.54	0.00037	1.50	0.00060	−2.44
cg04085542	1.00	0.00033	0.95	0.00094	−5.15
cg04162999	0.92	0.030	1.05	0.016	14.72
cg04342090	−1.55	0.0086	−1.46	0.014	−5.58
cg04775941	−1.67	0.020	−1.54	0.034	−7.5
cg04798929	2.20	0.0024	2.14	0.0034	−2.98
cg04983687	−1.18	0.000010	−1.43	0.0000017	21.45
cg05048002	−1.91	0.00074	−1.75	0.0036	−8.1
cg05104993	2.89	0.0018	3.18	0.0012	10.1
cg05560165	1.37	0.0011	1.55	0.00062	13.84
cg05652668	−1.15	0.00068	−1.27	0.00031	10.57
cg06816054	−1.37	0.0081	−1.49	0.0068	9.25
cg06824199	−2.11	0.000029	−2.45	0.000011	16.35
cg06851336	−1.78	0.00099	−1.76	0.0011	−0.86
cg07765167	−2.47	0.000065	−2.52	0.000095	1.94
cg07880854	1.21	0.014	1.43	0.0070	18.16
cg07908654	−1.67	0.000013	−1.80	0.0000099	7.96
cg07970948	−1.26	0.000027	−1.42	0.000014	12.22
cg08397758	0.84	0.043	1.90	0.0032	125.56
cg09249800	−1.14	0.000016	−1.27	0.0000094	11.06
cg09332506	−1.97	0.0000097	−2.10	0.0000078	6.38
cg09570585	0.85	0.064	0.82	0.082	−3.88
cg09635874	1.96	0.00037	1.90	0.0010	−3.11
cg10016610	−0.57	0.098	−0.63	0.085	9.8
cg11182893	1.81	0.00032	1.64	0.0015	−9.47
cg11372831	2.62	0.00018	2.63	0.00029	0.2
cg11569718	−1.54	0.011	−1.44	0.021	−6.64
cg12578575	−0.58	0.012	−0.59	0.014	1.49
cg12819826	−1.90	0.000087	−1.93	0.00029	1.67
cg12819873	−2.36	0.0000045	−2.47	0.0000051	4.97
cg13168187	1.65	0.0024	2.79	0.000043	69.09
cg13197551	−1.84	0.000062	−1.77	0.00016	−3.49
cg13233042	−1.64	0.000032	−1.74	0.000031	6.21
cg13713293	−1.43	0.00039	−1.40	0.00070	−1.67
cg14322298	−1.53	0.0031	−1.57	0.0052	2.77
cg14478663	1.81	0.00020	1.91	0.00017	5.64
cg14574726	1.75	0.0058	1.91	0.0037	9.54
cg15281774	−1.91	0.0014	−2.13	0.00076	11.37
cg17041511	−1.97	0.000021	−2.25	0.000027	14.69
cg17549513	1.62	0.0094	1.82	0.0051	12.16
cg17594242	1.72	0.000035	1.71	0.000071	−0.93
cg17971837	−3.38	0.000061	−3.64	0.000069	7.85
cg23527183	−0.83	0.0048	−0.92	0.0034	10.38
cg24077454	1.33	0.0056	1.27	0.012	−4.29
cg24836822	−1.09	0.000020	−1.17	0.000014	8.19
cg25854298	−1.40	0.000042	−1.51	0.000037	7.39
cg27202913	−0.67	0.010	−0.69	0.0094	2.01
cg27468224	−1.83	0.00013	−1.85	0.00019	1.29
cg27469152	−1.99	0.00021	−2.21	0.000094	11.21

Results of 62 logistic regressions between methylation M-values and atopy for each selected CpG. We present crude associations as well as associations adjusted for predicted cell proportions of CD8^+^ T cells, CD4^+^ T cells, natural killer cells, B-cells, monocytes, and granulocytes. β_1_ represents the value of the regression coefficient for the CpG site in that statistical model. The percent change in β-values (%Diff_β_) was calculated as [(crude β_1_ – adjusted β_1_)/crude β_1_] and was used to evaluate whether cell type influenced the selection of each CpG site

**Fig. 2:**
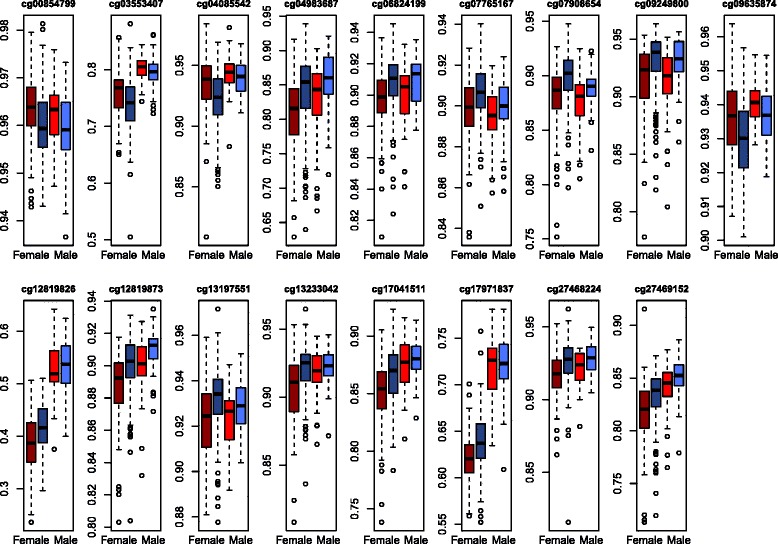
Distribution of methylation levels within the male and female samples, by atopy status. Boxplots showing the distribution of methylation levels within the male (*bright red* and *bright blue*) and female (*dark red* and *dark blue*) samples, stratified by atopy status (*red* = atopic; *blue* = non-atopic) among the 17 CpG sites significantly associated with atopy and present in both the male and female samples. The 30 sites that were not significant in stage 2 analyses are included in Additional file 1

Prior to running the stage 2 joint analyses we compared the distribution of methylation levels in the male and female samples stratified by atopy status. The distributions (Fig. 2 and Additional file 1) were similar between the two samples for most loci and thus we proceeded with pooling the data. However, since the distribution of methylation levels did differ by sex for some loci, we included sex as a covariate in the stage 2 analyses.

For stage 2, we tested each of the 62 CpG sites for their associations with atopy and high serum IgE levels in the joint sample. Only 50 of the 62 sites were present in both samples, because the female and male samples were analyzed as separate batches and 12 of these sites were removed from the male sample during data cleaning and pre-processing. The sites that were only present in the female sample were still analyzed in stage 2.

Of the 62 sites, 22 had statistically significant associations with atopy (*P*-value range 6.5E−9 to 7.9E−4) (Table 5). At four of these sites, higher levels of DNA-M were associated with increased likelihood of atopy [odds ratio (OR) range 2.66–8.08]. For the other 18 sites, lower levels of DNA methylation were associated with higher likelihood of atopy (OR range 0.311–0.065). We also found that 12 sites had statistically significant associations with both atopy and high IgE (*P*-value range 1.1E−5 to 7.1E−4) in the IOW. Set analyses [40] showed a mild but statistically significant joint effect of DNA methylation on atopy at the 10 IgE-associated and atopy-associated sites shared between men and women (estimate 0.0016, 95 % confidence interval 0.0003–0.023).

**Table 5:** Comparison of stage 2 (n = 367) and replication (n = 464) results

Genetic annotations^*^				IOW: atopy status^a^		IOW: high IgE^b^		BAMSE: atopy status^c^	
CpG Site	Chr	Gene name	CpG location	Adj. OR	*P-*Value	Adj. OR	*P-value*	Adj. OR	*P*-value
**cg04983687**	**16**	***ZFPM1***	**Body**	0.239	**6.46E−09**	0.158	**3.54E−05**	0.269	**3.17E−06**
cg09249800	1	*ACOT7*	Body	0.239	**8.52E−09**	0.141	**1.13E−05**	–	**–**
**cg12819873**	**11**	***PRG2***	**5′UTR**	0.065	**1.38E−08**	0.045	**7.37E−05**	0.140	**2.36E−05**
**cg07908654**	**13**	***–***	**–**	0.145	**1.14E−07**	0.103	**8.44E−05**	0.206	**2.21E−04**
**cg06824199**	**1**	***KIAA0494***	**Body**	0.107	**6.28E−07**	0.073	**1.93E−04**	0.118	**2.96E−06**
**cg27469152**	**17**	***EPX***	**3′UTR**	0.091	**1.34E−06**	0.025	**6.06E−05**	0.099	**1.00E−05**
**cg27468224**	**4**	***–***	**–**	0.123	**1.62E−06**	0.073	**5.01E−04**	0.262	**1.89E−02**
**cg13233042**	**11**	***ATL3***	**Body**	0.192	**5.06E−06**	0.105	**1.82E−04**	0.194	**9.75E−05**
**cg13197551**	**20**	***LSM14B***	**3′UTR**	0.160	**5.67E−06**	0.094	**5.64E−04**	0.312	**1.04E−02**
cg07765167	17	*MRPL45*	TSS1500	0.087	**6.50E−06**	0.043	**5.96E−04**	–	**–**
**cg09332506**	**1**	***COPA***	**Body**	0.123	**7.77E−06**	0.126	3.62E−03	0.196	**8.72E−05**
**cg17041511**	**17**	***–***	**–**	0.143	**1.27E−05**	0.075	8.14E−04	0.133	**4.31E−05**
cg24836822	7	*KCNH2*	Body	0.311	**1.36E−05**	0.225	**7.13E−04**	–	**–**
**cg07970948**	**7**	***ZNF862***	**Body**	0.243	**1.38E−05**	0.109	**2.55E−04**	0.313	**1.61E−05**
**cg25854298**	**10**	***ASCC1***	**Body**	0.221	**3.69E−05**	0.144	1.24E−03	0.149	**1.21E−06**
cg09635874	13	*FARP1*	Body	8.084	**4.89E−05**	2.591	2.44E−01	1.149	8.59E−01
cg04085542	5	*FAM172A*	Body; 5′UTR	2.663	**7.99E−05**	1.250	5.48E−01	0.936	7.93E−01
cg03553407	4	*ARHGAP10*	Body	4.330	**1.63E−04**	2.411	1.92E−01	2.010	2.16E−01
cg12819826	19	*PPAN; PPAN-P2RY11*	TSS1500	0.213	**2.45E−04**	0.229	4.02E−02	1.788	2.87E−01
cg00854799	1	*PEX10; RER1*	3′UTR	4.209	**2.48E−04**	2.930	8.27E−02	1.464	5.18E−01
cg05652668	7	*LUC7L2*	1st exon; 5′UTR	0.280	**3.07E−04**	0.517	2.39E−01	0.820	7.35E−01
**cg17971837**	**22**	***PVALB***	**TSS1500**	0.122	**7.90E−04**	0.102	4.11E−02	0.188	**2.60E−02**

Associations for methylation M-values with atopy and high IgE from the IOW epigenetic sample, as well as with atopy in the BAMSE replication sample. IOW analyses were adjusted for predicted cell proportions of CD8^+^ T cells, CD4^+^ T cells, natural killer cells, B cells, monocytes, granulocytes, and sex. BAMSE replication analyses were adjusted for the same predicted cell proportions, sex, batch (bisulfite treatment date), and asthma treatment

*Abbreviations: Adj. OR* adjusted odds ratio, *BAMSE* Children, Allergy, Milieu, Stockholm, Epidemiology cohort, *Chr* Chromosome number, *CpG* cytosine–phosphate–guanine, *IgE* Immunoglobulin E, *IOW* Isle of Wight cohort, *TSS* Transcription Start Site, *UTR* untranslated region

^*^CpGs and their annotations highlighted in bold were the sites replicated in the BAMSE cohort

^a^Atopy defined as at least one positive skin prick test; significant sites determined via α = 8.06E−4 (0.05/62 tests)

^b^High IgE defined as serum IgE ≥ 200 kU/L; significant sites determined via α = 8.06E−4 (0.05/62 tests)

^c^Atopy defined as serum IgE antibody ≥ 0.35 kU_A_/L, to any allergen; significant sites determined via α = 0.05

Finally, 19 of the 22 sites (data on three sites were not available in BAMSE) associated with atopy in IOW were studied in an independent cohort. Of the 19 sites tested, 13 were significantly associated (*P*-values < 0.05) with atopy in BAMSE and had comparable ORs (Table 5): cg04983687 in *zinc finger protein*, *FOG family member 1* (*ZFPM1*), cg18219873 in *proteoglycan 2* (*PRG2*), cg07908654 (intergenic), cg06824199 in *EF-hand calcium binding domain 14* (*KIAA0494*, also known as *EFCAB14*), cg27469152 in *eosinophil peroxidase* (*EPX*), cg27468224 (intergenic), cg13233042 in *atlastin GTPase 3* (*ATL3*), cg13197551 in *SCD6 homolog B* (*LSM14B,* also known as C20orf40), cg09332506 in *coatomer protein complex, subunit alpha* (*COPA*), g07970948 in *zinc finger protein 862* (*ZNF862*), cg25854298 in *activating signal cointegrator 1 complex subunit 1* (*ASCC1*), and cg17971837 in *parvalbumin* (*PVALB*). For all 13 sites, persons with atopy or high IgE had lower methylation levels compared to those without atopy or with lower IgE. All nine sites that were associated with both atopy status and high IgE in IOW after adjusting for multiple tests (*P*-value < 8.06E−4) were successfully replicated in BAMSE, whereas the remaining four replicated sites (cg09332506 in *COPA*, cg17041511 (intergenic), cg25854298 in *ASCC1*, and cg17971837 in *PVALB*) had nominal (*P*-value < 0.05) associations with high IgE in IOW. Interestingly, almost all sites (five out of six) that were not replicated in BAMSE did not have even nominal associations with high IgE in IOW.

The 13 replicated sites were investigated for functional annotation in DAVID and for individual biological relevance via literature review. Functional annotation of the 10 genes (*ZFPM1*, *PRG2*, *KIAA0494*, *EPX*, *ATL3*, *LSM14B*, *COPA, ZNF862, ASCC1*, and *PVALB*) associated with the 13 replicated CpG sites (Table 6) revealed three statistically significant annotations: polymorphism, eosinophil, and asthma. The most interesting of these findings involved two genes (*EPX* and *PRG2*) in the KEGG pathway for asthma (Benjamini *P*-value = 0.00056) and associated with the eosinophils annotation (Benjamini *P*-value = 0.0087).

**Table 6:** Functional annotation for genes associated with the 13 sites that were successfully replicated

Category	Term	Genes	*P*-value^*^
**SP_PIR_KEYWORDS**	**Polymorphism**	***COPA*** **,** ***KIAA0494*** **,** ***EPX*** **,** ***ZNF862*** **,** ***PRG2*** **,** ***ASCC1*** **,** ***ZFPM1***	**0.0145**
**SP_PIR_KEYWORDS**	**Eosinophil**	***EPX*** **,** ***PRG2***	**0.0087**
**KEGG_PATHWAY**	**Asthma**	***EPX*** **,** ***PRG2***	**0.0005**
UP_SEQ_FEATURE	Sequence variant	*COPA*, *KIAA0494*, *EPX*, *ZNF862*, *PRG2*, *ASCC1*, *ZFPM1*	0.1365
GOTERM_MF_FAT	GO:0005509 Calcium ion binding	*COPA*, *KIAA0494*, *PVALB*, *EPX*	0.1807
SP_PIR_KEYWORDS	Nitration	*EPX*, *PRG2*	0.1539
SP_PIR_KEYWORDS	Alternative splicing	*LSM14B*, *COPA*, *ZNF862*, *ATL3*, *ASCC1*	0.3382
GOTERM_MF_FAT	GO:0046872 Metal ion binding	*COPA*, *KIAA0494*, *PVALB*, *EPX*, *ZNF862*, *ZFPM1*	0.3749
GOTERM_BP_FAT	GO:0048193 Golgi vesicle transport	*COPA*, *ATL3*	0.8360
GOTERM_MF_FAT	GO:0043169 Cation binding	*COPA*, *KIAA0494*, *PVALB*, *EPX*, *ZNF862*, *ZFPM1*	0.2826
GOTERM_MF_FAT	GO:0043167 Ion binding	*COPA*, *KIAA0494*, *PVALB*, *EPX*, *ZNF862*, *ZFPM1*	0.2291
SP_PIR_KEYWORDS	Calcium	*KIAA0494*, *PVALB*, *EPX*	0.3469
SP_PIR_KEYWORDS	Phosphoprotein	*LSM14B*, *COPA*, *KIAA0494*, *PVALB*, *ATL3*, *ZFPM1*	0.2989
SP_PIR_KEYWORDS	Cytoplasmic vesicle	*COPA*, *PRG2*	0.5085
UP_SEQ_FEATURE	Splice variant	*LSM14B*, *COPA*, *ZNF862*, *ATL3*, *ASCC1*	0.9270
UP_SEQ_FEATURE	Domain:EF-hand 1	*KIAA0494*, *PVALB*	0.8342
UP_SEQ_FEATURE	Domain:EF-hand 2	*KIAA0494*, *PVALB*	0.7430
INTERPRO	IPR018249: EF-HAND 2	*KIAA0494*, *PVALB*	0.9655
GOTERM_BP_FAT	GO:0006350 Transcription	*ZNF862*, *ASCC1*, *ZFPM1*	0.9478
INTERPRO	IPR018247: EF-HAND 1	*KIAA0494*, *PVALB*	0.8227

Statistically significant DAVID functional annotation and pathway results are in bold

^*^ *P*-values corrected for multiple testing via Benjamini–Hochberg method

## Discussion

Our methodological approach and the biological relevance of our findings are noteworthy to researchers studying epigenetic mechanisms in atopy. We selected 62 CpG sites from a starting set of 254,460, resulting in vastly improved classification of atopics (from 78.95 to 14.47 % error) and non-atopics (from 19.87 % to 7.22 % error) when compared to the RF on the full dataset. Of particular note was the large proportion of CpG loci that were statistically significant at a Bonferroni-adjusted α for atopy (35 %) and high IgE (19 %) within the IOW sample and the large proportion (13 of 19) of sites that were successfully replicated in the BAMSE cohort.

Our findings are the latest in a series of recent work that supports the application of RF for genome-wide association studies (GWAS) and in allergic diseases. The recursive RF process we utilized was similar to methods proposed elsewhere [13, 15, 17, 35]. It has been used by Menze et al. [37] and Anaissi et al. [18] but, to the best of our knowledge, has never been implemented in epigenomics. Goldstein et al. presented one of the first successful applications of RF for GWAS, demonstrating its ability to identify genes known to be associated with the multiple sclerosis as well as genes with previously unknown disease associations [13]. Xu et al. successfully identified SNPs predictive of asthma exacerbations in children via RF [16]. These findings indicate the promising nature of the use of RF for feature selection in future epigenome-wide studies.

The true challenge with high-throughput techniques is in connecting the results to biological processes, which are complex and can involve combinations of many genes working together. We investigated the biological roles of the ten genes associated with the 13 replicated CpGs sites: *ZFPM1*, *PRG2*, *KIAA0494*, *EPX*, *ATL3*, *LSM14B*, *COPA, ZNF862*, *ASCC1*, and *PVALB*. For each of these genes, we performed a search of the literature for possible roles in atopy and conducted functional annotation in DAVID.

Among the replicated loci, a number of their associated genes were involved in intriguing processes that may have a role in atopy. *ZFPM1* (also known as *FOG-1*) is a binding factor for the transcription factor *GATA-1* and has been primarily studied for its role in the differentiation of erythroid, megakaryocyte, and mast cells [43]. However the consequences of *FOG-1* expression appear to be dependent on its cellular origin and the biochemical surroundings, which can determine whether *FOG-1* acts as a repressor or co-activator of *GATA-1* [43, 44]*.* Recently, *ZFPM1* was shown in an in vitro study to down-regulate IL-4 and therefore facilitate T_H_1 differentiation [45]. Also, two differentially methylated regions in *ZFPM1* were recently identified in association with asthma [46]. The multifaceted roles of *ZFPM1* in immune-cell activity and allergic disease suggest that this is an interesting yet possibly overlooked gene in atopy and atopic diseases.

Eosinophils are subtypes of granulocytes that are heavily involved in inflammatory responses and atopic asthma through the mechanism of airway inflammation [47]. *EPX* encodes eosinophil peroxidase, a protein expressed by eosinophils. Previous investigations found that both serum and urine levels of EPX were elevated in children who had positive SPTs, as well as those with allergic diseases such as asthma, allergic rhinoconjunctivitis and atopic dermatitis [48–51]. A recent epigenome-wide study found multiple CpG sites, including one within *PRG2*, which were associated with high versus low total IgE, primarily driven by eosinophils. Interestingly, this study also found that the methylation levels in isolated eosinophils differed among asthmatics with high total IgE, asthmatics with low total IgE, and controls, suggesting that eosinophils from persons with allergic hypersensitivity or asthma may have different epigenetic profiles compared to eosinophils from non-allergic individuals [52]. Also, a recent genome-wide expression study of peripheral blood mononuclear cells found that *PRG2* expression was up-regulated in response to dust-mite exposure, suggesting a possible role in the adaptive immune response [53].

A GWAS of atopic asthma implicated SNPs that were in linkage disequilibrium with SNPs in *COPA*, though these did not achieve genome-wide significance [54]. More recently, four deleterious variants within *COPA* have been linked to an autoimmune disease characterized by high-titer autoantibodies, interstitial lung disease, and inflammatory arthritis [55]. These mutations may induce stress on the endoplasmic reticulum leading to defective intracellular protein transport between the golgi and the endoplasmic reticulum; such defects have been linked to autoimmune and lung-disease. Interestingly, mutant *COPA* also appears to drive CD4^+^ T-cells toward T_H_17 phenotype via increased expression of IL-1β, IL-6 and IL-23 [55]. Thus, mutant COPA does appear to affect immune pathways which can lead to autoimmune disease and our findings suggest that differential epigenetic regulation of *COPA* may play a role in hypersensitivity, though further research is necessary to elucidate this role.

*LSM14B* may be involved in mRNA translation [56, 57]. Some of the genes encode proteins that perform structural roles in different areas of the body. ATL3 participates in tethering, creating a tubular connective network of membranes in the endoplasmic reticulum, which is the site where ribosomes build proteins from DNA transcripts. The functional annotation results implicated the genes *EPX* and *PRG2* in eosinophil activity and in the KEGG pathway for late hypersensitive responses in asthma. Some of the genes (*KIAA0494*, *ATL3*, *LSM14B*, *ASCC1*, and *PVALB*) did not have any apparent role in immune response.

These findings should be interpreted within the limitations of the study. Although we provide evidence in support of associations between 13 CpG sites and atopy, variations in methylation at these sites may not cause allergic sensitization. The cross-sectional nature of this sample prohibited us from distinguishing between which DNA-M variations at CpG sites may have caused, been caused by, or just been markers of sensitization. However, associations in any of these directions may yield important insights into the development, persistence, and consequences of allergic sensitization. Some of the CpG sites that were selected could not be replicated and some that were replicated were not involved in any known biological processes related to atopy or allergy. The unsuccessful replication could be due to false-positive findings from the discovery analyses, or differences in how atopy was assessed between the discovery and replication cohorts. The lack of biological roles for these CpG sites could be explained by selected CpG sites possibly being highly correlated with other CpG sites that truly influence atopy status, or by CpG sites having roles in unknown, but still important, biological pathways involved in atopy.

Correlated predictors may present an issue that we were unable to address [13, 17]. If the methylation level at a biologically important CpG site was highly correlated with methylation levels at other unimportant loci, the inclusion of those unimportant loci in a forest would decrease the VIM of the important CpG site and may result in its exclusion during data reduction. This would result in a statistically strong but biologically ambiguous result. It is possible that some of our results that were not biologically consistent with allergic disease were due to this issue. Applying an approach similar to linkage disequilibrium and haplotype identification from genetic studies may improve the prediction accuracy of the forest and save computational time [17], but such applications have not been studied with genome-wide DNA-M arrays at this time. Furthermore, there is no consensus with respect to which VIM is best for large-scale data with correlated predictors. We used MDG, which was also utilized by Menze et al. to recursively eliminate unimportant predictors [37]. Calle and Urrea found that MDA was unstable when there were small alterations to the data, but that MDG was robust to such changes [36]. However, MDG does not perform as well if the scales of the variables differ widely or if they have different numbers of categories [36, 58], which would be an important consideration for researchers incorporating both DNA-M and SNP data in a single dataset. More work needs to be done to determine which VIMs perform best under the typical characteristics of genome-wide DNA-M studies: sparsity, skewed continuous predictors, very large *n* and very small *p*, statistical interactions, or correlations between predictors. Despite the issues of correlated predictors, such variables can still provide useful information. DNA-M loci that are merely surrogates of actual CpGs associated with atopy can still serve as biomarkers of disease, but do not serve to improve our understanding of the etiology of atopy.

Some of the CpGs that we identified with the recursive RF but that did not meet our replication criteria may in fact be biologically relevant in atopy. We would not expect all biologically relevant findings to be included in the functional annotation results of our gene list for two reasons: first, our gene list of 10 genes is quite small because DAVID is optimized for lists between 100 and 2,000 genes in length [41]; and second, functional annotation relies on current knowledge of gene functions, and may not correctly classify the functions of novel loci. Also, these sites were selected with RF, which allows for complex interactions to be identified [13]. CpGs that were selected via RF due to unknown interaction effects may not have had an independent association with atopy and thus could not have survived our stage 2 analyses with strict multiple testing adjustments to significance levels.

Despite correcting for cell proportions (CD8^+^ T cells, CD4^+^ T cells, natural killer cells, B cells, monocytes, and granulocytes) in our regression analyses, the predicted cell proportions for the low-frequency cell types, such as T-cell subtypes, may be less accurate than those of the higher frequency cell types and these predictions did not distinguish eosinophils from other granulocytes. Given the importance of T-cell subtypes (T_H_1, T_H_2, and T_H_17) and eosinophils (a subset of granulocytes) in atopic responses, this may have resulted in some residual confounding. However, given the inability to collect actual cell differentials in this study, the predictions we used likely accounted for the majority of cellular heterogeneity in our blood samples.

The lack of independence between the samples used for RF feature selection (stage 1) and the samples used for determinations of statistical significance (stage 2) was another limitation, and may have led to some over-fitting during stage 2 analyses. Also, 12 CpG sites that were selected in stage 1 were not present in the male sample. Although these were still evaluated in the stage 2 analyses, the lack of full methylation data reduced the power to identify significant findings at these 12 sites. However, the strong replication results in the BAMSE cohort would suggest that the majority of our findings were not due to random chance or over-fit to the IOW sample.

Not all of our findings were replicated; six sites that were tested did not successfully replicate and three sites could not be tested because the data were unavailable. The six non-replicated sites may represent false-positives from our stage 2 analyses or could be due to differences in the measurement of atopy status between the two cohorts. One limitation of the replication study was that atopy was defined as at least one positive SPT to any allergen in the IOW; whereas atopy was defined as specific IgE antibody ≥ 0.35 kU_A_/L to any allergen in BAMSE. The associations with high serum IgE in the IOW support that at least some of the unsuccessful replications may have been due to these differences in measurement. All 13 sites that replicated in BAMSE had at least nominal associations with high serum IgE in IOW (*P*-values < 0.05), whereas only one of the six sites that did not replicate in BAMSE had an association with high serum IgE in IOW (*P*-value < 0.05). These findings suggest that the only sites that could be replicated in this study may be involved in IgE-mediated allergic sensitization. Also, some atopy-associated CpG sites in IOW, which were measured at 18 years old, may not have been able to replicate in BAMSE, measured at 8 years of age, because methylation levels can be age dependent [59]. It is possible that some of these six sites may have replicated had the outcome of atopy status been measured with the same method and at the same age in both cohorts. Thus, although these six sites were not considered positive findings in this study, future epigenetic studies that utilize SPTs to evaluate sensitization, and evaluate sensitization in young adults close to age 18, may consider attempting to replicate these sites. The three sites for which data were not available in the replication cohort should also be considered for future replication studies. The CpG site (cg09249800) in *ACOT7*, which was strongly associated with both atopy and high IgE in the IOW cohort, is particularly interesting because others have identified differentially methylated regions within this gene associated with asthma [46]; thus, it may play a role in allergic sensitization or allergic diseases.

## Conclusions

Utilizing a two-stage design with a well-characterized but sparsely implemented RF feature selection method followed by logistic regression for both atopy and an alternate marker of atopy (high IgE), we identified a number of CpG sites associated with atopy. Most importantly, 13 sites were replicated in an independent cohort for atopy status: cg04983687 in the body of *ZFPM1*, cg12819873 in the 5′UTR of *PRG2*, cg07908654 (intergenic), cg06824199 in the body of *KIAA0494*, cg27469152 in the 3′UTR of *EPX*, cg27468224 (intergenic), cg13233042 in the body of *ATL3*, cg13197551 in the 3′UTR of *LSM14B*, cg09332506 in the body of *COPA*, cg17041511 (intergenic), cg07970948 in the body of *ZNF862*, cg25854298 in the body of *ASCC1*, and cg17971837 in the TSS1500 of *PVALB*. Three of the 22 sites associated with atopy in IOW were not available for testing in the BAMSE cohort, so may be of interest for follow-up in future studies of DNA-M and atopy: cg09249800 in the body of *ACOT7*, cg07765167 in the TSS1500 of *MRPL45*, and cg24836822 in the body of *KCNH2*. These CpG sites and their associated genes could be treated as under-studied candidates for future studies of atopy; particularly cg04983687 in *ZFPM1*, cg12819873 in *PRG2,* cg27469152 in *EPX*, and cg09332506 in *COPA*. Furthermore, we showed that recursive RF data reduction can be an effective approach for epigenome-wide DNA-M studies, and may be considered by other investigators as it has now been successful in multiple studies with large-scale data.

## Additional file

Additional file 1:
**Boxplots of the distributions of methylation levels within the male (** ***bright red*** **and** ***bright blue*** **) and female (** ***dark red*** **and** ***dark blue*** **) samples, stratified by atopy status (** ***red*** **= atopic;** ***blue*** **= non-atopic) for the 33 CpGs sites present in both the male and female samples that were not significantly associated with atopy in stage 2 analyses.** (PDF 20 kb)

## Abbreviations

BAMSE: Children, Allergy, Milieu, Stockholm, Epidemiology (Swedish abbreviation)
CpG: cytosine-phosphate-guanine
DNA-M: DNA methylation
GWAS: genome-wide association study
IgE: immunoglobulin E
IOW: Isle of Wight
MDA: mean decrease accuracy
MDG: mean decrease Gini
OOB-ER: out-of-bag error rate
OR: odds ratio
QC: quality control
RF: Random Forest
SNP: single nucleotide polymorphism
SPT: skin prick test
VIM: variable importance measures
