# Supplementary figures and images for: DNA methylation loci associated with atopy and high serum IgE: a genome-wide application of recursive Random Forest feature selection
Source: Genome Med. 2015 Aug 21;7(1):89. doi: 10.1186/s13073-015-0213-8 (PMC4545869; doi:10.1186/s13073-015-0213-8)

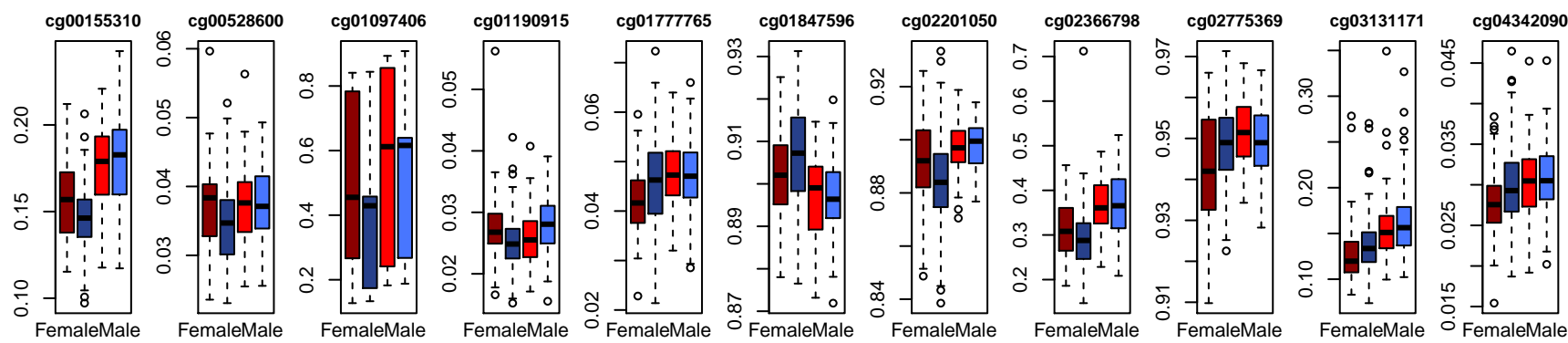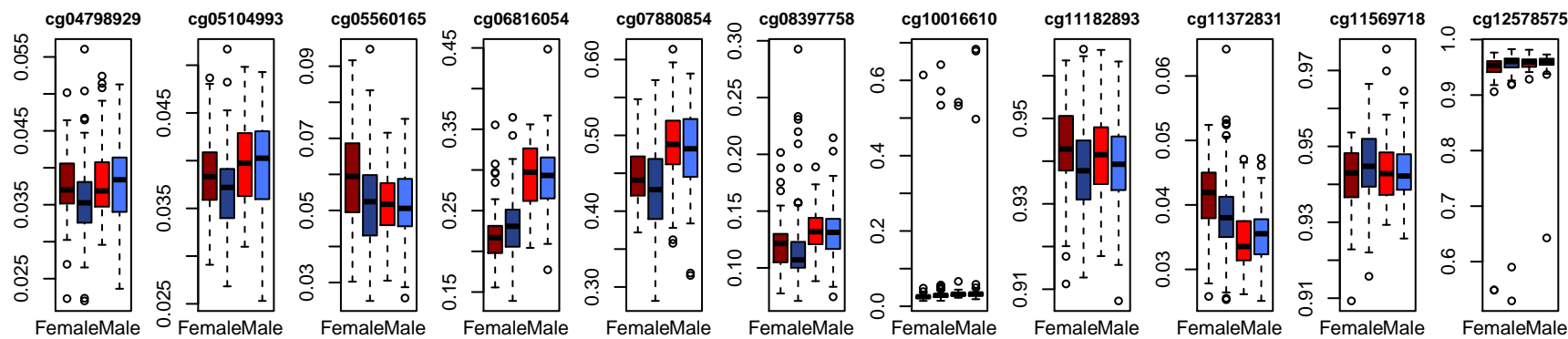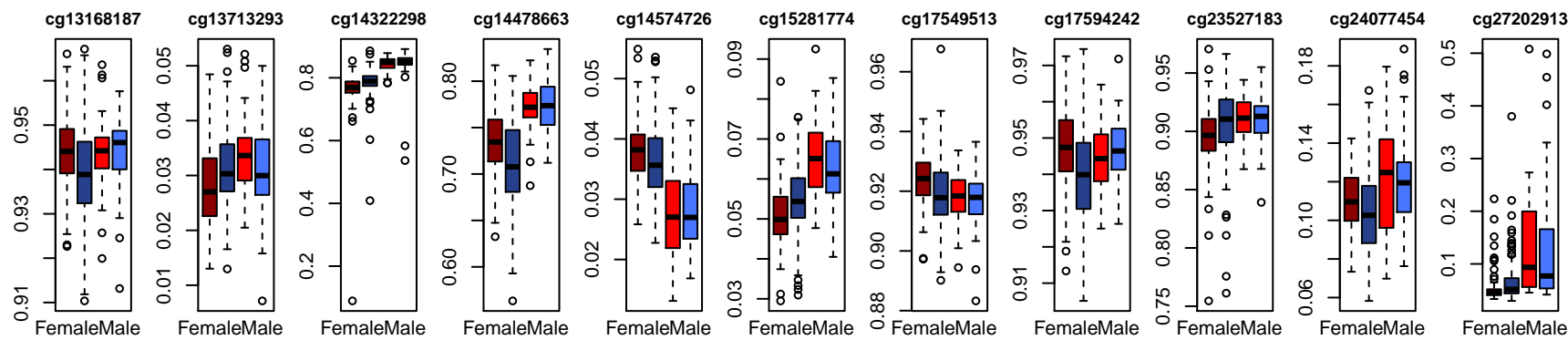

Supplement: Additional file 1: — Boxplots of the distributions of methylation levels within the male ( bright red and bright blue ) and female ( dark red and dark blue ) samples, stratified by atopy status ( red = atopic; blue = non-atopic) for the 33 CpGs sites present in both the male and female samples that were not significantly associated with atopy in stage 2 analyses. (PDF 20 kb) [file 13073_2015_213_MOESM1_ESM.pdf]
